# Supplementary material for: Genetics and environment distinctively shape the human immune cell epigenome
Source: Nat Genet. 2026 Jan 27;58(2):392–403. doi: 10.1038/s41588-025-02479-6 (PMC12900638; doi:10.1038/s41588-025-02479-6)
Supplement: Supplementary file 1 — Reporting Summary [file 41588_2025_2479_MOESM1_ESM.pdf]

nature portfolio

Corresponding author(s): Joseph R. Ecker

Last updated by author(s): Oct 31, 2025

Reporting Summary

Nature Portfolio wishes to improve the reproducibility of the work that we publish. This form provides structure for consistency and transparency in reporting. For further information on Nature Portfolio policies, see our Editorial Policies and the Editorial Policy Checklist.

Statistics

For all statistical analyses, confirm that the following items are present in the figure legend, table legend, main text, or Methods section.

n/a	Confirmed	
☐	☒	The exact sample size (*n*) for each experimental group/condition, given as a discrete number and unit of measurement
☐	☒	A statement on whether measurements were taken from distinct samples or whether the same sample was measured repeatedly
☐	☒	The statistical test(s) used AND whether they are one- or two-sided<br>*Only common tests should be described solely by name; describe more complex techniques in the Methods section.*
☐	☒	A description of all covariates tested
☐	☒	A description of any assumptions or corrections, such as tests of normality and adjustment for multiple comparisons
☐	☒	A full description of the statistical parameters including central tendency (e.g. means) or other basic estimates (e.g. regression coefficient) AND variation (e.g. standard deviation) or associated estimates of uncertainty (e.g. confidence intervals)
☐	☒	For null hypothesis testing, the test statistic (e.g. F, t, r) with confidence intervals, effect sizes, degrees of freedom and P value noted<br>*Give P values as exact values whenever suitable.*
☒	☐	For Bayesian analysis, information on the choice of priors and Markov chain Monte Carlo settings
☒	☐	For hierarchical and complex designs, identification of the appropriate level for tests and full reporting of outcomes
☐	☒	Estimates of effect sizes (e.g. Cohen's *d*, Pearson's *r*), indicating how they were calculated

Our web collection on statistics for biologists contains articles on many of the points above.

Software and code

Policy information about availability of computer code

Data collection: Codes of all the analysis are available on github (https://github.com/wangwl/ECHO) and Zenodo (10.5281/zenodo.17307293).

Data analysis:
Software and algorithms
bismark (v0.20.0) (Krueger and Andrews, 2011) https://github.com/FelixKrueger/Bismark
Trim Galore (4.4) https://www.bioinformatics.babraham.ac.uk/projects/trim_galore/; RRID:SCR_011847
samtools (Li et al., 2009) http://www.htslib.org/
subread (Liao et al., 2013) https://subread.sourceforge.net/
Picard MarkDuplicates (Version:3.0.0) https://broadinstitute.github.io/picard/
ALLCools (1.1.1) https://github.com/lhqing/ALLCools
snapATAC2 (2.9.0.dev0) (Fang et al., 2021;
Zhang et al., 2021)
https://github.com/kaizhang/SnapATAC2
methylpy (1.4.6) (Schultz et al., 2016)
https://github.com/yupenghe/methylpy
GREAT (McLean et al., 2010;
Tanigawa et al., 2022) http://great.stanford.edu/public/html/index.php
R (4.3.1) https://cran.r-project.org; RRID:SCR_001905
MACS3 (3.0.3b) (Zhang et al., 2008) https://github.com/taoliu/MACS
Homer (v5.1) (Heinz et al., 2010) http://homer.ucsd.edu/homer; RRID:SCR_010881
bedtools (v2.31.1) https://bedtools.readthedocs.io/en/latest/#; RRID:SCR_006646
wigToBigWig (v2.9) http://hgdownload.cse.ucsc.edu/admin/exe/

April 2023

Metascape (v3.5) (Zhou et al., 2019) https://metascape.org/gp/index.html#/main/step1; RRID:SCR_016620
QTLtools (v2.0-7-g61a04d2c5e) https://qtltools.github.io/qtltools/
coloc (v5.2.3) https://cran.r-project.org/web/packages/coloc/index.html
smr (v1.0)https://yanglab.westlake.edu.cn/software/smr/#Overview

For manuscripts utilizing custom algorithms or software that are central to the research but not yet described in published literature, software must be made available to editors and reviewers. We strongly encourage code deposition in a community repository (e.g. GitHub). See the Nature Portfolio guidelines for submitting code & software for further information.

Data

Policy information about availability of data

All manuscripts must include a data availability statement. This statement should provide the following information, where applicable:

- Accession codes, unique identifiers, or web links for publicly available datasets
- A description of any restrictions on data availability
- For clinical datasets or third party data, please ensure that the statement adheres to our policy

De-identified molecular data and associated sample metadata generated in this study are available through controlled access via the Database of Genotypes and Phenotypes (dbGaP) under accession number phs003204.v1.p1. Access to these data is subject to approval by the dbGaP Data Access Committee in accordance with NIH policies on the sharing of human genomic data.
The single-cell ATAC-seq data generated and analyzed in this study have been deposited in the NCBI Gene Expression Omnibus (GEO) under accession number GSE306525.
All other data supporting the findings of this study are available within the article and its Supplementary Information files. Source data are provided with this paper.

Research involving human participants, their data, or biological material

Policy information about studies with human participants or human data. See also policy information about sex, gender (identity/presentation), and sexual orientation and race, ethnicity and racism.

Reporting on sex and gender	We have included both male and female sexes in our analysis wherever the samples were available for the various exposures.
Reporting on race, ethnicity, or other socially relevant groupings	Self-reported ethnicity, age, sex etc where available for most of the exposure samples we have analyzed. This is provided as Supplementary information.
Population characteristics	For most exposures, the donors are from a broad and mixed population.
Recruitment	We did not recruit any volunteers for the exposure samples. These were provided by sample provider collaborator sites through their respective separate studies.
Ethics oversight	The work was conducted after Salk Institutional Review Board (IRB) approval through IRB Protocol Number: 18-0015 titled "Single Cell Analysis for Forensic Epigenetics (SAFE)." Salk Federal Wide Assurance (FWA) for the Protection of Human Subject Number: FWA00005316.

Note that full information on the approval of the study protocol must also be provided in the manuscript.

Field-specific reporting

Please select the one below that is the best fit for your research. If you are not sure, read the appropriate sections before making your selection.

☒ Life sciences ☐ Behavioural & social sciences ☐ Ecological, evolutionary & environmental sciences

For a reference copy of the document with all sections, see nature.com/documents/nr-reporting-summary-flat.pdf

Life sciences study design

All studies must disclose on these points even when the disclosure is negative.

Sample size	Previous studies performed in our lab and others have shown that methylation differences using chi-square based tests between cases and control samples can provide signficance levels of at least 0.05
Data exclusions	No data is excluded
Replication	Biological replicates including both sexes were used.
Randomization	Randomization details are available for each exposure, respectively.
Blinding	Investigators have access only to alphanumerical IDs linked to basic metadata.

Reporting for specific materials, systems and methods

We require information from authors about some types of materials, experimental systems and methods used in many studies. Here, indicate whether each material, system or method listed is relevant to your study. If you are not sure if a list item applies to your research, read the appropriate section before selecting a response.

Materials & experimental systems

n/a	Involved in the study
☒	☐ Antibodies
☒	☐ Eukaryotic cell lines
☒	☐ Palaeontology and archaeology
☒	☐ Animals and other organisms
☒	☐ Clinical data
☒	☐ Dual use research of concern
☒	☐ Plants

Methods

n/a	Involved in the study
☒	☐ ChIP-seq
☐	☒ Flow cytometry
☒	☐ MRI-based neuroimaging

Plants

Seed stocks: *Report on the source of all seed stocks or other plant material used. If applicable, state the seed stock centre and catalogue number. If plant specimens were collected from the field, describe the collection location, date and sampling procedures.*

Novel plant genotypes: *Describe the methods by which all novel plant genotypes were produced. This includes those generated by transgenic approaches, gene editing, chemical/radiation-based mutagenesis and hybridization. For transgenic lines, describe the transformation method, the number of independent lines analyzed and the generation upon which experiments were performed. For gene-edited lines, describe the editor used, the endogenous sequence targeted for editing, the targeting guide RNA sequence (if applicable) and how the editor was applied.*

Authentication: *Describe any authentication procedures for each seed stock used or novel genotype generated. Describe any experiments used to assess the effect of a mutation and, where applicable, how potential secondary effects (e.g. second site T-DNA insertions, mosiacism, off-target gene editing) were examined.*

Flow Cytometry

Plots

Confirm that:

☒ The axis labels state the marker and fluorochrome used (e.g. CD4-FITC).

☒ The axis scales are clearly visible. Include numbers along axes only for bottom left plot of group (a 'group' is an analysis of identical markers).

☒ All plots are contour plots with outliers or pseudocolor plots.

☒ A numerical value for number of cells or percentage (with statistics) is provided.

Methodology

Sample preparation: CELL THAWING/COUNTING: Quick thaw cells in 37°C water bath, Pipette cells into 5 mL PBS, rinse tube with 1 mL PBS, Spin 5 min, aspirate supernatant, Resuspend in 10 mL PBS, Count, Aliquot and spin 5 min, aspirate supernatant
STAINING: Zombie dye – 1:100 dilution in PBS, Resuspend cells in 66 µL of Zombie dilution, Incubate 10 min @ RT, covered, Add 5 µL Human TruStain FcX to sample, Incubate 10 min @ RT, covered; Ab cocktail: 3 µL x 8 Abs, 5 µL CCR7, Add 29 µL cocktail to sample, Incubate 15-20 min @ RT, covered
WASH: Add 1 mL PBS/2% FBS, Spin 5 min, aspirate supernatant, Resuspend in 1 mL PBS/2% FBS, Spin 5 min, aspirate supernatant
FIX: Resuspend in 500 µL FluoroFix Buffer (1% PFA), Incubate 30 min @ RT, Add 800 µL PBS/2% FBS, Spin 5 min, aspirate supernatant, Resuspend in 1 mL PBS/2% FBS, Store at 4°C until sort

Instrument: BD Influx and Sony MA900

Software: FlowJo and Sony Cell Sorter Software Version 3.1.2

Cell population abundance: Figure S1B

Gating strategy: Figure S1A

☒ Tick this box to confirm that a figure exemplifying the gating strategy is provided in the Supplementary Information.
